# Heme-based catalytic properties of human serum albumin

**DOI:** 10.1038/cddiscovery.2015.25

**Published:** 2015-09-07

**Authors:** P Ascenzi, A di Masi, G Fanali, M Fasano

**Affiliations:** 1 Interdepartmental Laboratory for Electron Microscopy, Roma Tre University, 00146 Roma, Italy; 2 Department of Sciences, Roma Tre University, 00146 Roma, Italy; 3 Biomedical Research Division, Department of Theoretical and Applied Sciences, University of Insubria, 21052 Busto Arsizio, Italy; 4 Center of Neuroscience, University of Insubria, 21052 Busto Arsizio, Italy

## Abstract

Human serum albumin (HSA): (i) controls the plasma oncotic pressure, (ii) modulates fluid distribution between the body compartments, (iii) represents the depot and carrier of endogenous and exogenous compounds, (iv) increases the apparent solubility and lifetime of hydrophobic compounds, (v) affects pharmacokinetics of many drugs, (vi) inactivates toxic compounds, (vii) induces chemical modifications of some ligands, (viii) displays antioxidant properties, and (ix) shows enzymatic properties. Under physiological and pathological conditions, HSA has a pivotal role in heme scavenging transferring the metal-macrocycle from high- and low-density lipoproteins to hemopexin, thus acquiring globin-like reactivity. Here, the heme-based catalytic properties of HSA are reviewed and the structural bases of drug-dependent allosteric regulation are highlighted.

## Facts

- Human serum albumin (HSA) is the most abundant protein in plasma.
- HSA binds endogenous and exogenous ligands at multiple sites.
- HSA has a pivotal role in heme scavenging.
- Human serum heme-albumin (HSA-heme) displays metal-based multienzymatic properties.
- Heme-based catalytic properties of HSA are modulated allosterically by endogenous and exogenous ligands, including drugs, representing a pivotal issue in the pharmacological therapy management.

## Open questions

- In spite of the multifaced roles of HSA, humans almost totally lacking this protein safely survive. Are HSA actions essential?
- *α*-Fetoprotein, the fetal homolog of HSA, is recognized by its specific receptor. Does a specific receptor also occur for human serum albumin?
- HSA-heme may represent a heme-based enzyme and a blood substitute.

HSA, the most represented plasma protein (~7.5×10^−4^ M), (i) modulates the plasma oncotic pressure, (ii) regulates fluid distribution between the body compartments, (iii) represents a depot and a transporter of endogenous and exogenous compounds (e.g., fatty acids (FA), metal ions, drugs, hormones, toxins and metabolites), (iv) increases the apparent solubility and lifetime of hydrophobic compounds, (v) affects pharmacokinetics of many drugs, (vi) induces chemical modifications of some ligands (e.g., drugs), (vii) inactivates some toxic compounds, (viii) displays antioxidant properties, and (ix) shows enzymatic properties.^1,2^

Under physiological and pathological conditions, HSA has a pivotal role in heme scavenging.^3,4^ In fact, although heme regulates gene expression and is the prosthetic group of heme proteins under physiological conditions, high levels of free heme: (i) catalyze the synthesis of toxic free hydroxyl radicals, (ii) affect the integrity of erythrocyte membranes, (iii) induce the enrollment to the vascular endothelium of red blood cells, platelets, and leukocytes, and (iv) cause the oxidation of low-density lipoproteins.^5–9^ HSA takes out heme from high- and low-density lipoproteins and transfers the metal-macrocycle to hemopexin. After endocytosis of the hemopexin-heme complex into the hepatic parenchymal cells through the CD91 receptor, hemopexin releases heme intracellularly. After heme delivery, hemopexin is released intact into the bloodstream, and the heme is degraded.^3,4,10^ Therefore, HSA acquires time-dependent globin-like properties, representing a case for ‘chronosteric effects’.^2,11^ Interestingly, heme-based catalytic properties of HSA are modulated allosterically by drugs, representing a pivotal issue in the pharmacological therapy management.^2^

Here, the heme-based catalytic properties of HSA are reviewed and the structural bases of drug-dependent allosteric regulation are highlighted.

## The HSA structural organization

HSA is an all-*α*-helical protein consisting of three structurally similar domains usually indicated as I (amino acids 1–195), II (amino acids 196–383), and III (amino acids 384–585). The three domains of HSA assemble asymmetrically and resemble a heart shape. Each domain includes 10 *α*-helices that are packed in two separate subdomains (named A and B) comprising six (h1–h6) and four (h7–h10) *α*-helices, respectively; the subdomains are connected by a long extended loop. Terminal regions of the sequential domains contribute to the formation of interdomain regions, 9-turn-long *α*-helices link domain IB to IIA (residues 173–205) and IIB to IIIA (residues 336–398). The HSA fold is stabilized by 17 intrasubdomain disulfide bridges.^2^

**Figure 1:**
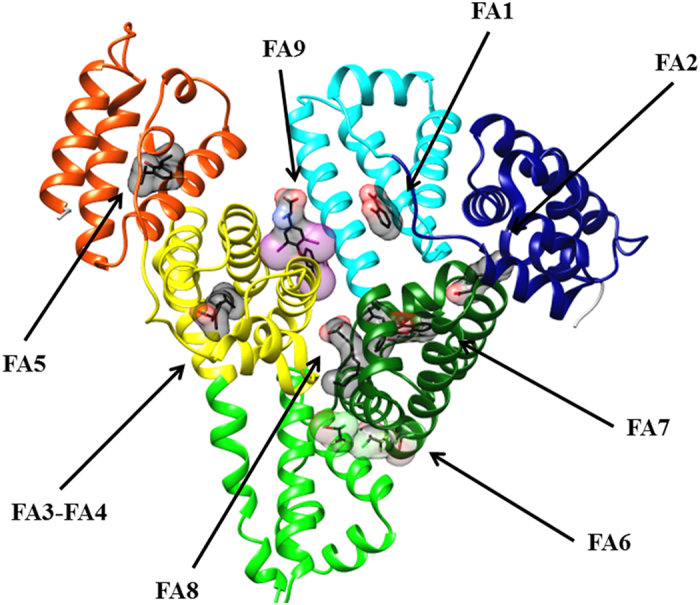
Three-dimensional structure of HSA complexed with endogenous and exogenous ligands bound to the FA sites. The subdomains of HSA are rendered with different colors (domain IA, in blue; domain IB, in cyan; domain IIA, in forest green; domain IIA, in green; domain IIIA, in yellow; domain IIIB, in orange). The FA1-salicylic acid (PDB ID: 2I30),^36^ F2-capric acid (PDB ID: 1E7E),^17^ FA3-FA4-ibuprofen (PDB ID: 2BXG),^15^ FA5-propofol (PDB ID: 1E7A),^35^ FA6-halothane (PDB ID: 1E7C),^35^ FA7-warfarin (PDB ID: 2BXD),^15^ F8-capric acid (PDB ID: 1E7E),^17^ and FA9-thyroxine (PDB ID: 1HK4)^39^ complexes are highlighted. Three molecules of halothane are bound in the FA6 site. The picture has been drawn with the UCSF Chimera package.^94,95^

The multidomain organization of HSA is at the root of its extraordinary ligand-binding ability and capacity.^1,2,12^ The most relevant clefts hosting ligands (e.g., heme, FAs, bilirubin, and drugs) are the so-called FA binding sites (named FA1 to FA9). Bacterial protein recognition cleft(s), thyroxine-binding sites, and metal ion recognition sites also participate to HSA actions.^1,2,12–16^ (Figure 1).

The FA1 binding site (located in subdomain IB) binds very different ligands (e.g., heme, bilirubin, FAs, and exogenous compounds) and has been reported to represent a major drug-binding pocket. Upon ligand binding to the FA1 site, a conformational change(s) propagates from subdomain IB to subdomains IA, IIA, and IIB.^15,17–20^ Heme binds to HSA by the IA–IB loop. In particular, Tyr138 and Tyr161 form *π*–*π* stacking interactions with the macrocycle ring, the Tyr161 side chain being coordinated to the heme-Fe atom by the phenoxyl O atom, and the Arg114, His146, and Lys190 residues being involved in salt bridges with the heme propionates.^21^ In contrast, bilirubin binding to FA1 does not need *π*–*π* stacking by Tyr138 and Tyr161 for ligand recognition.^22^ The FA carboxylate head-group is hydrogen bonded to Arg117 and to a water molecule, which is in turn stabilized by hydrogen bonding to the phenoxyl and carbonyl O atoms of Tyr161 and Leu182, respectively.^17^ Lastly, Tyr138 and Tyr161 have a key role also in drug recognition.^18–20^

The FA2 site (located at the interface between subdomains IA, IB, and IIA) has been postulated to represent a secondary recognition cleft of ibuprofen and warfarin.^2,17,23–26^ The FA carboxylate head-group is bound to subdomain IIA through Tyr150, Arg257, and Ser287, whereas the methylene tail is placed in the hydrophobic cleft between subdomains IA and IIA.^17^ FAs binding to FA2 stabilize the B-conformation of HSA and promote the ligand-induced conformational transition(s).^2,17,23–26^

The FA3–FA4 cleft (located in subdomain IIIA) is also named Sudlow’s site II and represents a major drug-binding pocket. The FA3–FA4 cleft binds preferentially aromatic carboxylates showing an extended conformation, with ibuprofen representing the prototypical ligand.^2,15,27–34^ Drugs (e.g., ibuprofen) cluster in the center of the FA3–FA4 cleft, interacting with the hydroxyl group of Tyr411 and forming salt bridges and hydrogen bond interactions with Arg410 and Ser489 residues. The FA carboxylate head-group binds to Ser342 and Arg348 placed in the subdomain IIB and to the Arg485 side chain sited in the subdomain IIIA. In the FA4 site, the FA carboxylate head-group is bound to Arg410, Tyr411, and Ser489 placed externally in the subdomain IIIA, whereas the hydrophobic tail is accommodated into the subdomain IIIA.^15,17,34^

The FA5 site (positioned in subdomain IIIB) accommodates FAs whose carboxylate head-groups bind to Tyr401 and Lys525.^2,15,17,31,35^

The FA6 site (placed between subdomains IIA and IIB) represents the secondary binding site of diflunisal, halothane, and ibuprofen.^2,15,35^ The FA carboxylate head-group binds to Arg209, Lys351, and Ser480, whereas the methylene tail contacts Arg209, Asp324, and Glu354.^17,36^

The FA7 site (placed in subdomain IIA) is also named Sudlow’s site I and represents a major drug-binding pocket. This pocket binds preferentially heterocyclic compounds, with warfarin representing the prototypical ligand.^1,2,15^ The FA7 site is also the primary binding site for 3-carboxy-4-methyl-5-propyl-2-furanpropanoic acid, azapropazone, di-iodosalicylic acid, indomethacin, iodipamide, oxyphenbutazone, phenylbutazone, and tri-iodobenzoic acid, and represents the secondary binding cleft for diflunisal and indoxyl sulfate.^15^ The FA7 cleft also houses one aspirin or two salicylic acid molecules.^36^ Drugs (e.g., warfarin) cluster in the center of the FA7 site, having a planar group pinned snugly between the apolar side chains of Leu238 and Ala291 and making a hydrogen bond interaction with the hydroxyl group of Tyr150. Moreover, warfarin forms hydrogen bonds with His242, and with either Lys199 or Arg222. The FA7 site recognizes FAs by salt bridging the FA carboxylate head-group to Arg257.^15,17,31,34,37^ Lastly, HSA can covalently bind different FA7 ligands because of the presence of Lys199, which can act as a nucleophile.^38^

FA8, taking place upon the FA-induced conformational transition(s), is located at the base of the gap between subdomains IA, IB, and IIA on one side and subdomains IIB, IIIA, and IIIB on the other side. Owing to volume restrictions, FA8 only binds short-chain FAs. The Lys195, Lys199, Arg218, Asp451, and Ser454 residues form an open ring that stabilizes the FA carboxylate head-group.^17^

FA9, occurring upon the FA-induced conformational transition(s), lies in an upper region of the interdomain region paved by subdomains IA, IB, and IIA on one side and subdomains IIB, IIIA and IIIB on the other side. A salt bridge between Glu187 of domain I and Lys432 of domain III contributes to keep the FA in place.^17,39^

Binding of the thyroid hormone T4 to HSA is modulated allosterically. In FA-free HSA, the thyroid hormone T4 binds to four sites (i.e., Tr-1 to Tr-4). In particular, the Tr-1 site (corresponding to the FA7 site) is positioned in the subdomain IIA, the Tr-2 site (overlapping the FA3–FA4 cleft) is located in the subdomain IIIA, and the Tr-3 and Tr-4 sites (matching the FA5 site) are placed in the subdomain IIIB. Of note, two T4 molecules are accommodated in the FA5 pocket. Although in the presence of myristate binding of the thyroid hormone T4 to the Tr-1 to Tr-4 sites of HSA is impaired, this FA induces conformational change(s) leading to the opening of the fifth T4-binding pocket (i.e., Tr-5), which is located between domains I and III and partially corresponds to the FA9 site. Remarkably, the Arg218 mutation within subdomain IIA greatly enhances the affinity of HSA for the thyroid hormone T4 causing the increase of the serum thyroxine level, which is associated with familial dysalbuminemic hyperthyroxinemia.^39^

HSA binds several endogenous and exogenous proteins,^40^ including the protein-G-related albumin-binding module of the anaerobic bacterium *Finegoldia magna* poly(A)-binding protein in the proximity of the FA6 site.^41,42^ This protein provides selective advantages to the bacterium, delivering FAs and other nutrients transported by HSA.^41,43^

HSA displays a wide variety of binding sites for several metal ions, including Mg(II), Al(III), Ca(II), Mn(II), Co(II/III), Ni(II), Cu(I/II), Zn(II), Cd(II), Pt(II), Au(I/II), Hg(II), and Tb(III).^1,16,44–47^ Three major binding sites endowed with appropriate residues matching for the different metal ligand fields have been observed.^16^ The first site (called the N-terminal binding site) is located at the N-terminus, where Cu(II), Co(II), and Ni(II) are coordinated by nitrogen donor atoms from Asp1, Ala2, and His3.^48^ The second site is represented by the free Cys34 thiol that binds Au(I), Hg(II), and Pt(II) ions.^1,49^ The third metal-binding site (called the primary multimetal binding site or cadmium site A) involves His67, Asn99, His247, and Asp249 residues. Owing to its pseudo-octahedral geometry, this site stably allocates different metal ions, representing the primary cleft for Zn(II) and Cd(II) and the secondary site of Cu(II) and Ni(II).^50^ The secondary site for Cd(II) binding (called as secondary multimetal binding site or cadmium site B) has not been firmly identified.^51^

## Allosteric modulation by drugs of heme-based catalytic properties of HSA

The heme-based catalytic properties of HSA are strictly dependent on the molar fraction of HSA-heme present in the plasma. In fact, because of the low plasma level of HSA-heme in healthy subjects (1.5×10^−6^ M), the heme-based catalytic properties of HSA appear to be relevant, especially in patients affected with hematologic diseases showing high intravascular hemolysis and high HSA-heme plasmatic levels (reaching 5×10^−5^ M).^25,52^ The heme-based catalytic properties of HSA are modulated allosterically by drugs, and, according to linked functions,^53^ the oxidation state and the axial coordination of the heme-Fe atom affect drug recognition.^2^

### Peroxynitrite scavenging by HSA-heme-Fe(II)-NO

Peroxynitrite reacts with HSA-heme-Fe(II)-NO leading to HSA-heme-Fe(III) and NO, according to Scheme 1.^54^

In the absence and presence of CO_2_, values of *k*_on_ for peroxynitrite scavenging by ferrous nitrosylated HSA-heme, horse heart myoglobin, and human hemoglobin are grossly similar; however, they are lower compared with those reported for ferrous nitrosylated rabbit hemopexin-heme and human neuroglobin by about one order of magnitude (Table 1).^54–57^ Remarkably, abacavir promotes allosterically peroxynitrite scavenging by HSA-heme-Fe(II)-NO; in fact, the *k*_on_ value increases from 6.5×10^3^/M/s, in the absence of the drug, to 2.2×10^4^/M/s, in the presence of saturating amounts of abacavir.^54^ In addition, CO_2_ accelerates peroxynitrite scavenging by ferrous nitrosylated heme proteins mediated by about one order of magnitude (Table 1).^54–57^ This reflects the transient formation of the highly reactive CO_3_^●−^ species following the reaction of peroxynitrite with CO_2_.^58–60^ In particular, the value of *k*_on_ for peroxynitrite detoxification by ferrous nitrosylated HSA-heme increases from 6.5×10^3^/M/s, in the absence of CO_2_, to 1.3×10^5^/M/s, in the presence of CO_2_.^54^ Moreover, abacavir and CO_2_ cooperate in peroxynitrite scavenging by HSA-heme-Fe(II)-NO, with the value of *k*_on_ rising to 3.6×10^5^/M/s.^54^

In the absence and presence of CO_2_, the rate-limiting step of peroxynitrite detoxification by ferrous nitrosylated heme proteins is represented by NO dissociation from the heme(III)-NO adduct. Moreover, values of *h*_off_ are CO_2_-independent, spanning over two orders of magnitude; of note, the value of *h*_off_ for the denitrosylation of the HSA-heme-Fe(III)-NO complex (1.8×10^−1^/s) is unaffected by abacavir (Table 1).^54–57^

As a whole, peroxynitrite scavenging with the concomitant release of NO may switch peroxynitrite signaling to NO-based strategies opening new avenues in cell metabolism.

### Peroxynitrite detoxification by HSA-heme-Fe(III)

HSA-heme-Fe(III) catalyzes peroxynitrite detoxification according to Scheme 2.^24,61–63^

Values of *l*_on_ for peroxynitrite detoxification by ferric mammalian heme proteins range from 1.2×10^4^ to 4.3×10^5^/M/s (Table 2).^24,64–67^ Peroxynitrite detoxification by HSA-heme-Fe(III) prevents free L-Tyr nitration, possibly displaying a protective role *in vivo*. Chlorpropamide, digitoxin, furosemide, ibuprofen, imatinib, indomethacin, isoniazid, phenylbutazone, rifampicin, sulfisoxazole, tolbutamide, and warfarin impair allosterically peroxynitrite isomerization in a dose-dependent manner. Indeed, the value of *k*_on_ for peroxynitrite detoxification by HSA-heme-Fe(III) decreases from 4.3×10^5^/M/s, in the absence of drugs, to 3.5×10^4^/M/s, in the presence of saturating amounts of chlorpropamide, digitoxin, furosemide, ibuprofen, imatinib, indomethacin, isoniazid, phenylbutazone, rifampicin, sulfisoxazole, tolbutamide, and warfarin. The drug-dependent allosteric inhibition of peroxynitrite detoxification by HSA-heme-Fe(III) promotes free L-Tyr nitration, thus modulating protein actions, structure, and metabolism.^24,61–63^ However, peroxynitrite detoxification by ferric mammalian heme proteins is unaffected by CO_2_, which in turn facilitates the spontaneous isomerization of peroxynitrite to the harmless NO_3_^−^.^24,64–67^

Interestingly, ibuprofen affects to the same extent peroxynitrite detoxification by both full-length HSA-heme-Fe(III) and truncated HSA-heme-Fe(III) devoided of domain III (Table 2), indicating that domains I and II form the allosteric core of HSA.^67^

As a whole, the drug-dependent modulation of peroxynitrite detoxification catalyzed by HSA-heme-Fe(III) may affect indirectly signaling pathways involving NO and O_2_^●−^,^24,61–63^ which are the precursors of peroxynitrite.^59^

### The HSA-heme-Fe(II)-catalyzed conversion of NO_2_^−^ to NO

HSA-heme-Fe(II) catalyzes the conversion of NO_2_^−^ to NO under anaerobic conditions, according to Scheme 3.^68^

Values of *b*_on_ for NO_2_^−^ conversion to NO_2_^−^ by mammalian heme proteins range between 1.2×10^−2^ and 6.0/M/s (Table 3), reflecting the different structural and chemical features of the heme site.^68–73^ In particular, the low *b*_on_ values for the conversion of NO_2_^−^ to NO by ferrous human cytoglobin could reflect the hexa coordination of the heme-Fe(II) atom.^73^ Moreover, the reactivity of ferrous human neuroglobin reflects the reversible redox-linked hexa- to penta-coordinate transition of the heme Fe(II) atom. In fact, under oxidative conditions, the formation of the Cys46–Cys55 disulfide bridge stabilizes the highly reactive penta-coordinate heme-Fe(II) atom facilitating the reaction, whereas under reductive conditions the cleavage of the Cys46–Cys55 bridge leads to the formation of the low-reactivity hexa-coordinate heme-Fe(II) atom.^72^ Furthermore, the ferrous HSA-heme-Fe(II)- and human hemoglobin-catalyzed conversion of NO_2_^−^ to NO is impaired allosterically by warfarin and inositol hexakisphosphate, respectively.^68–70^ In particular, the *b*_on_ value for the ferrous HSA-heme-Fe(II)-catalyzed conversion of NO_2_^−^ to NO decreases from 1.3/M/s, in the absence of warfarin, to 0.1/M/s at saturating drug concentration.^68^

The nitrite reductase activity of mammalian heme proteins may have a relevant role in acidosis and anoxia; in fact, *b*_on_ increases on pH decrease and on NO_2_^−^ concentration increase, under anaerobic conditions.^68–73^

### O_2_-based scavenging of NO-bound HSA-heme-Fe(II)

HSA-heme-Fe(II)-NO is irreversibly oxidized by O_2_ with the concomitant production of harmless NO_3_^−^, according to Scheme 4.^74^

The NO dissociation rate constant (i.e., *d*_off_ ) represents the rate-limiting step of the O_2_-mediated oxidation of HSA-heme-Fe(II)-NO.^74^ Remarkably, this reaction is modulated allosterically by rifampicin, which accelerates the dissociation of NO. In fact, the value of *d*_off_ increases from 9.8×10^−5^/s, in the absence of the drug, to 8.8×10^−4^/s, in the presence of saturating amounts of the drug.^74^ This behavior is reminiscent of that reported for the O_2_-mediated oxidation of ferrous nitrosylated human hemoglobin, wherefore inositol hexakisphosphate affects the reaction by shifting the quaternary equilibrium; consequently, values of *d*_off_ increase from 3.2×10^−4^ and 7.0×10^−5^/s in the R-state to 2.5×10^−3^ and 9.8×10^−5^/s in the T-state.^75^ In contrast, O_2_ reacts directly with ferrous nitrosylated horse heart myoglobin and rabbit hemopexin-heme, and this leads to the formation of the transient peroxynitrite-bound ferric heme protein species, which precedes the appearance of the final products, that are, ferric heme protein and nitrate.^74,76,77^ Lastly, a slight rearrangement within the protein structure, which may take place after the formation of ferric human neuroglobin, has been postulated to be the rate-limiting step in O_2_-mediated oxidation of ferrous nitrosylated neuroglobin.^56^

### Catalase and peroxidase activity of HSA-heme-Fe(III)

HSA-heme-Fe(III) exhibits catalase and peroxidase activity in the oxidation of phenolic compounds related to Tyr (i.e., *p*-cresol, 3-(*p*-hydroxyphenyl)propionic acid, tyramine, and tyrosine) and 2,20-azinobis(3-ethylbenzothiazoline-6-sulfonate) according to Scheme 5.^78,79^ In Scheme 5, HSA-heme-Fe(III)* is the active HSA-heme-Fe(III), S is the substrate, HSA-heme-Fe(III)-S is the HSA-heme-Fe(III) substrate adduct, HSA-heme-Fe(III)*-S is the active HSA-heme-Fe(III) substrate adduct, P is the product, *f*_1_ is the rate constant for the formation of active species, *f*_1_′ is the rate constant for the transformation of HSA-heme-Fe(III)-S into HSA-heme-Fe(III)*-S, *f*_p_ is the rate constant for product formation, *f*_c_ and *f*_c_′ are the rate constants for the decomposition of peroxide by HSA-heme-Fe(III)-S and HSA-heme-Fe(III)*-S, respectively, and *F*_b_ and *F*_b_′ are the dissociation equilibrium constants for substrate (i.e., S) binding to HSA-heme-Fe(III) and HSA-heme-Fe(III)*, respectively.

In the minimum reaction mechanism depicted by Scheme 5, the binding processes were considered fast with respect to the reaction processes. Values of *f*_1_ and of the *f*_p_/*f*_c_′ ratio, which rule the competition between catalase and peroxidase activity of HSA-heme-Fe(III), range between 4.1×10^1^ and 1.6×10^2^/M/s, and between 5.3×10^−3^ and 6.3×10^−2^ M, respectively, depending on the substrate.^78,79^

The catalytic mechanism of HSA-heme-Fe(III) appears to be different from that of peroxidases. In fact, the rate of formation of the active HSA-heme-Fe(III) species (i.e., HSA-heme-Fe(III)*) is lower compared with its reaction rate with either the substrate (in a peroxidase reaction) or the peroxide (in a catalase reaction) or the heme-Fe(III) itself. The weak catalase and peroxidase activity of HSA-heme-Fe(III) reflects the reduced accessibility of the heme-Fe(III) center, and the lack of an Arg residue in the HSA-heme-Fe(III) pocket that in peroxidases assists the cleavage of bound peroxide and accelerates the formation of the active species.^78^

## Structural bases of HSA allostery

The three-domain structure of HSA is at the root of its allosteric features, which are associated with pH changes, as well as with ligand binding. It is worthy noting that the allosteric modulation of HSA reactivity by drugs is relevant *in vivo* and represents a pivotal issue in the pharmacological therapy management.^2^

Domains I and II form the allosteric core of HSA, with the FA1, FA2, FA6, and FA7 sites being functionally linked. In fact, the functional properties of wild-type HSA are superimposable to those of truncated HSA, which contains only domains I and II.^67,80^ Moreover, contacts between subdomains IA and IIA have been reported to be pivotal in the allosteric modulation of HSA actions.^81^ Therefore, the C-terminal domain III of HSA, containing the FA3–FA4 and the FA5 sites, has a minor role in the allosteric modulation of ligand-binding and reactivity properties.^2,67,80,82–84^ However, (i) FAs modulate allosterically and competitively ligand binding to HSA involving domains I, II, and III^85^, and (ii) the allosteric linkage between the FA3–FA4 cleft (in domain III) and the FA7 site (in domain II) affects benzodiazepines, warfarin, and phenprocoumon enantiomers binding.^86,87^

**Figure 2:**
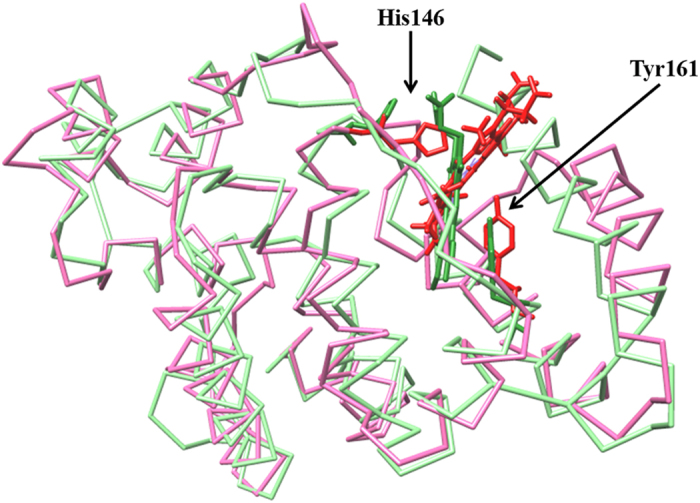
Drug-dependent six coordination of the heme-Fe atom of HSA-heme. Superposition of the crystal structure of HSA-heme-Fe(III) (light green and forest green, PDB entry 1O9X)^91^ and of the binary drug-bound HSA-heme-Fe(III) complex (hot pink and red).^26^ Heme-Fe(III), His146 and Tyr161 are highlighted. The picture has been drawn using the UCSF Chimera package.^94,95^

Drugs, including ibuprofen and warfarin, modulate allosterically the catalytic properties of HSA-heme affecting the coordination state of the heme-Fe atom. The catalytically active species of HSA-heme display a four- or five-coordinated heme-Fe atom, whereas the HSA-heme-inactive form shows a six-coordinated heme-Fe atom. In particular, Tyr161 coordinates the heme-Fe atom in the five-coordinated species, whereas Tyr161 and His146 are the fifth and sixth coordination ligands of the metal center in the six-coordinated species. Upon drug binding to HSA-heme (most probably to the FA2 site, the sole binding pocket contacting domains I and II), the reorientation of the Glu131-Arg145 *α*-helix and the axial coordination of the heme-Fe atom by His146 occur; as a consequence, the unreactive six-coordinated HSA-heme species becomes predominant. It is worth noting that the sixth His-Fe coordination bond of six-coordinated HSA-heme is ~2.15 Å in agreement to that observed in heme proteins^2,23,24,26,61–63,67,81,88–92^ (Figure 2).

## Conclusion

Several pathological conditions such as hematological diseases are characterized by altered heme plasma levels, which in turn switch HSA to HSA-heme and induce heme-based catalysis; this is particularly relevant considering the detoxification role of HSA-heme. Moreover, the heme-based catalytic properties of HSA are modulated allosterically by drugs. In turn, the oxidation state and the axial coordination of the heme-Fe atom affect drug recognition. This represents a pivotal issue in the pharmacological therapy management; in fact, drug association could inhibit allosterically the heme-based catalytic properties of HSA-heme (e.g., the detoxification of reactive nitrogen and oxygen species). Lastly, the heme-based catalytic properties of HSA are time-dependent reflecting the heme shuttling by HSA from low- and high-density lipoproteins to hemopexin, thus representing a case of ‘chronosteric effects’.

## Figures and Tables

**Figure:**
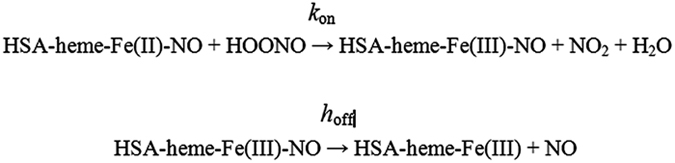
Scheme 1 Peroxynitrite scavenging by HSA-heme-Fe(II)-NO.

**Figure:**
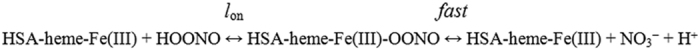
Scheme 2 Peroxynitrite detoxification by HSA-heme-Fe(III)

**Figure:**
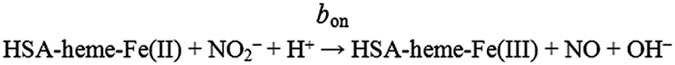
Scheme 3 The HSA-heme-Fe(II)-catalyzed conversion of NO_2_^–^ to NO.

**Figure:**
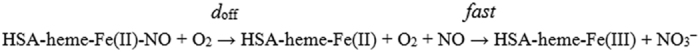
Scheme 4 O_2_-based scavenging of NO-bound HSA-heme-Fe(II).

**Figure:**
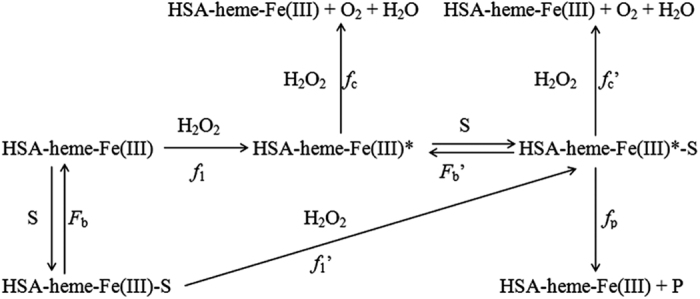
Scheme 5 Catalase and peroxidase activity of HSA-heme-Fe(III).

**Table 1:** Peroxynitrite scavenging by ferrous nitrosylated mammalian heme proteins

*Heme protein*	*[CO*_*2*_*] (M)*	*k*_*on*_ *(/M/s*)	*h*_off_ *(/s*)
Horse heart myoglobin	—a	3.1×10^4^^a^	~1.2×10^1^^a^
	1.2×10^−3^^b^	1.7×10^5^^b^	1.1×10^1^^b^
			
Human neuroglobin^c^	—	1.3×10^5^	1.2×10^−1^
HSA-heme^d^	—	6.5×10^3^	1.9×10^−1^
	1.2×10^−3^	1.3×10^5^	1.7×10^−1^
			
Abacavir-HSA-heme^d^	—	2.2×10^4^	1.8×10^−1^
	1.2×10^−3^	3.6×10^5^	1.9×10^−1^
			
Human hemoglobin^e^	—	6.1×10^3^	~1
	1.2×10^−3^	5.3×10^4^	~1
			
Rabbit hemopexin-heme^f^	—	8.6×10^4^	4.3×10^−1^
	1.2×10^−3^	1.2×10^6^	4.3×10^−1^

a pH 7.5 and 20.0 °C.^57^

b pH 7.0 and 20.0 °C.^57^

c pH 7.2 and 20.0 °C.^56^

d pH 7.0 and 10.0 °C.^54^

e pH 7.2 and 20.0 °C.^55^

f pH 7.0 and 10.0 °C.^93^

**Table 2:** Peroxynitrite scavenging by ferric mammalian heme proteins

*Heme protein*	*l_on_ (/M/s*)
Horse heart myoglobin^a^	2.9×10^4^
Sperm whale myoglobin^b^	1.6×10^4^
Human hemoglobin^c^	1.2×10^4^
Human HSA-heme^d^	4.1×10^5^
Ibuprofen-human HSA-heme e	3.5×10^4^
Truncated human HSA-heme f	4.3×10^5^
Ibuprofen-truncated human HSA-heme g	5.8×10^4^
Cardiolipin-horse heart cytochrome *c* h	3.2×10^5^

a pH 7.0 and 20.0 °C.^64^

b pH 7.5 and 20.0 °C.^65^

c pH 7.5 and 20.0 °C.^64^

d pH 7.2 and 22.0 °C.^24^

e pH 7.2 and 22.0 °C. Ibuprofen was 1.0×10^–2^ M.^24^

f pH 7.0 and 20.0 °C.^67^

g pH 7.0 and 20.0 °C. Ibuprofen was 1.0×10^–2^ M.^67^

h pH 7.0 and 20.0 °C. Cardiolipin was 1.6×10^−4^ M.^66^

**Table 3:** Ferrous mammalian heme-protein-catalyzed conversion of NO_2_^−^ to NO

*Heme protein*	**b**_*on*_ *(/M*/*s*)
Horse heart myoglobin^a^	2.9
Sperm whale myoglobin^b^	6.0
Mouse neuroglobin^c^	5.1
Human cytoglobin^d^	1.4×10^−1^
Human neuroglobin Cys46–Cys55^e^	1.2×10^−1^
Human neuroglobin Cys46/Cys55^f^	1.2×10^−2^
HSA-heme^g^	1.3
Warfarin-HSA-heme^g^	9.3×10^−2^
*Human hemoglobin*^b^	
T-state	1.2×10^−1^
R-state	6.0
Horse hearth cytochrome *c*^h^	7.0×10^−2^

a pH 7.4 and 25.0 °C.^72^

b pH 7.4 and 25.0 °C.^70^

c pH 7.4 and 25.0 °C.^71^

d pH 7.0 and 25.0 °C.^73^

e pH 7.4 and 25.0 °C. In human neuroglobin Cys46–Cys55, the Cys46 and Cys55 residues form an intramolecular disulfide bond.^72^

f pH 7.4 and 25.0 °C. In human neuroglobin Cys46/Cys55, the Cys46 and Cys55 residues do not form an intramolecular disulfide bond.^72^

g pH 7.4 and 20.0 °C.^68^

h pH 7.4 and 25.0 °C.^73^

## Abbreviations

FA: fatty acid
HSA: human serum albumin
HSA-heme: human serum heme-albumin.
